# Application of nitric oxide measurements in clinical conditions beyond asthma

**DOI:** 10.3402/ecrj.v2.28517

**Published:** 2015-08-17

**Authors:** Andrei Malinovschi, Dora Ludviksdottir, Ellen Tufvesson, Giovanni Rolla, Leif Bjermer, Kjell Alving, Zuzana Diamant

**Affiliations:** 1 Department of Medical Sciences: Clinical Physiology, Uppsala University, Uppsala, Sweden; 2 Department of Respiratory Medicine and Sleep, Landspitali University Hospital, Reykjavik, Iceland; 3 Department of Respiratory Medicine and Allergology, Institute for Clinical Science, Lund University, Lund, Sweden; 4 Department of Medical Sciences, Allergology and Clinical Immunology, University of Torino, Torino, Italy; 5 Department of Women's and Children's Health, Uppsala University, Uppsala, Sweden; 6 Department of Clinical Pharmacy & Pharmacology, University Medical Centre Groningen, Groningen, The Netherlands; 7 Department of General Practice, University Medical Centre Groningen, Groningen, The Netherlands; 8 QPS Netherlands, Groningen, The Netherlands

**Keywords:** exhaled nitric oxide, allergic rhinitis, COPD, cystic fibrosis, bronchopulmonary dysplasia, respiratory infections, pulmonary hypertension, hepatopulmonary syndrome, scleroderma, allograft rejections

## Abstract

Fractional exhaled nitric oxide (FeNO) is a convenient, non-invasive method for the assessment of active, mainly Th2-driven, airway inflammation, which is sensitive to treatment with standard anti-inflammatory therapy. Consequently, FeNO serves as a valued tool to aid diagnosis and monitoring in several asthma phenotypes. More recently, FeNO has been evaluated in several other respiratory, infectious, and/or immunological conditions. In this short review, we provide an overview of several clinical studies and discuss the status of potential applications of NO measurements in clinical conditions beyond asthma.

In asthma, fractional exhaled nitric oxide (FeNO) is a well-established, non-invasive online method for the assessment of active, mainly T-helper 2 (Th2) -driven airway inflammation, often associated with airway eosinophilia, which is sensitive to treatment with inhaled corticosteroids (ICSs) (1). In the past two decades, this method has been further refined and portable equipment has been developed and validated against chemiluminescence devices (2). Consequently, for more than a decade, FeNO has been implicated as a valued tool in the diagnosis and monitoring of corticosteroid-sensitive asthma.

More recently, FeNO has been evaluated in several other respiratory, allergic, infectious, and/or immunological conditions. In this short review, we provide an overview of studies applying FeNO measurements in several clinical conditions (Table 1) and underlying pathophysiological and biological mechanisms. In addition, potential clinical applications of NO measurements and future needs beyond asthma will be discussed.

**Table 1:** Summary of nitric oxide measurements in different clinical conditions

	FeNO	CalvNO	nNO
Asthma	Normal/increased	Normal/increased	Normal/increased
COPD	Normal/increased	Increased	NS
CF	Normal/decreased	Contradictory results	Decreased
PCD	Normal/decreased	Contradictory results	Decreased
Allergic rhinitis	Normal/increased	Normal	NS
BPD	Normal/decreased	Decreased	NS
HPS	Decreased	Increased	NS
PAH	Decreased	Increased	NS
SSc	Normal/decreased	Increased	NS
Rhinovirus infection	Increased	NS	NS
Allograft rejection	Increased	NS	NS

NS: not studied, COPD: chronic obstructive pulmonary disease, CF: cystic fibrosis, PCD: primary ciliary dyskinesia, BPD: bronchopulmonary dysplasia, HPS: hepatopulmonary syndrome, PAH: pulmonary arterial hypertension, SSc: systemic sclerosis.

## NO in chronic obstructive pulmonary disease

Persistent airway inflammation in both large and small airways is a hallmark of chronic obstructive pulmonary disease (COPD), causing progressive airflow limitation, airway remodeling, and dyspnea. Several studies have underscored the importance of the underlying systemic inflammation in the pathophysiology of COPD (3–5). The presence of concomitant systemic inflammation seems to be related to disease severity and frequency of exacerbations and can be measured as an increment of circulating cytokines (IL-6, TNF-*α*), chemokines (IL-8), and acute phase proteins, such as C-reactive protein (CRP) (6–8). In line with the chronic inflammation, increased expression of inducible nitric oxide synthase (iNOS) has been demonstrated in both central and peripheral airways of COPD patients (9). FeNO levels in unselected COPD patients are not different from healthy controls, whereas subgroups with increased FeNO levels have been reported (10). Patients with severe COPD have overall lower FeNO levels than patients with milder disease (11). In addition, smoking history must be taken into account as smoking affects FeNO levels. Current smokers with COPD had significantly lower FeNO levels compared to ex-smoking COPD patients (12). Peripheral airway inflammation in COPD can be reflected by increased alveolar NO levels (CalvNO) (13, 14). Surprisingly, CalvNO was not suppressed by systemic or ICSs in ultrafine particles (15).

In asthma, FeNO has been regarded as a marker of ICS responsive, Th2–driven airway inflammation, often associated with airway eosinophilia, with increased FeNO levels during asthma exacerbations. In COPD, exacerbations often follow respiratory infections that can be reflected by increases in FeNO levels (16) and systemic inflammation markers (17). In a recent study about ex-smoking COPD patients, FeNO was shown to correlate with sputum eosinophilia during exacerbations, and hence acted as predictor of ICS-responsiveness in these patients (18). In several COPD studies, FeNO had only a weak predictive role to detect improvement in symptoms or lung function after starting treatment with corticosteroids (12, 19, 20). An analysis of published controlled randomized studies using blood eosinophils as an algorithm to treat COPD exacerbations with prednisolone showed that a blood eosinophil count ≥2% was associated with a significantly better response to systemic prednisolone compared to a blood eosinophil count <2% (21).

More recently, a subgroup of patients with asthma COPD overlap syndrome (ACOS) has gained attention as it is now recognized that these patients have more frequent and more severe exacerbations (22). This COPD phenotype also has less emphysema, increased airway wall thickening, with often strongly variable FEV_1_ and non-specific airway hyperresponsiveness (23). ACOS has been associated with polymorphism in the gene *GPR65* (23), which is associated with eosinophil activation. So far, FeNO has not been specifically studied in ACOS and such studies are needed to understand the value of FeNO measurements in this phenotype.

Based on recent data, FeNO may have a role in the phenotyping and monitoring of distinct COPD subsets, including in predicting corticosteroid responsiveness in ACOS patients and those with eosinophilic airway inflammation (24). Implementation of FeNO measurements in the management of COPD needs further investigation.

## NO in cystic fibrosis

Cystic fibrosis (CF) is characterized by a defect in a chloride ion channel across the respiratory epithelium, among other organs, that leads to increased mucus viscosity, accumulation of mucus, chronic infection, and inflammation. FeNO levels are reported to be decreased in CF, with further decreases following a mouthwash (25). The degree of chloride ion transport impairment appears to relate to the decrease in FeNO levels (26).

The lower NO production is probably related to the reduction of NO production as both constitutional (27, 28) and inducible NOS (29, 30) activity is reduced. Furthermore, asymmetric dimethylarginine (ADMA), an endogenous NO synthase inhibitor, is increased in the sputum of CF patients (31), whereas arginine, a substrate for NO production, is reduced (32). Another FeNO-decreasing mechanism in CF might be an increased catabolism of NO (33). Lower levels of FeNO can also be due to mechanical factors as increased secretions might reflect a diffusion barrier for NO. Reduced airway caliber might relate to a lower bronchial epithelial area in contact with airway lumen and therefore lower FeNO (34). Furthermore, increased secretions might cause a diffusion barrier and further reduce exhaled NO. Previous animal studies argued for a role of NO deficiency in impaired airway relaxation in CF which might contribute to the airway obstruction (35). Interestingly, the low FeNO levels seen in CF can be further reduced by systemic corticosteroids (36).

In CF, supplementation with L-arginine by infusion (37), oral administration (38), or inhalation (39, 40) resulted in increased FeNO levels. Antibiotic treatment resulted in increased FeNO levels in one study (41), but this was not reproduced in another study, although a subgroup of patients appeared to exhibit an increase in FeNO levels (42). Dornase alpha treatment did not significantly affect FeNO in a small-scale study (43), but changes in FeNO levels related to changes in lung function in dornase alpha-treated-CF patients.

Several contradictory reports on levels of CalvNO in CF patients have been published (44, 45). CalvNO appeared to relate to ventilatory efficiency during exercise and airway obstruction in one study (46), but more studies are needed to confirm these findings.

Even if there is evidence for decreased levels of FeNO in CF patients with a link toward at least lower lung function, there is still lack of data regarding the role of repeated FeNO measurements, for example, in the annual follow-up or follow treatment with antibiotics in CF. Presently, there is no evidence for a role for CalvNO measurements in the management of CF.

## NO in bronchopulmonary dysplasia

Prematurity and its main respiratory complication, bronchopulmonary dysplasia (BPD), are potentially associated with lifelong lung function abnormalities, but the inflammatory component is most prominent during the neonatal period. Preterm infants with BPD show a weak postnatal increase in FeNO on day 28 as well as at a postmenstrual age of 49 weeks, particularly in those developing moderate or severe BPD (47, 48). Children with BPD are at increased risk for respiratory symptoms, including wheeze, cough, and dyspnea, even though the inflammatory component often diminishes during the first years. In a follow-up study of a subgroup of 53 previously preterm children (aged 10±1.5 years, 28 with BPD) 60% wheezed at age <2 years, but only 13% wheezed in the past year. In addition, non-specific airway hyperresponsiveness was present in 49% despite normal FeNO levels (49). At school age, children with very low birth weight (with or without BPD) showed lung function abnormalities characterized by airway obstruction, hyperinflation, and diffusion impairment, with reduced or normal FeNO levels (50–53). CalvNO levels in schoolchildren with BPD were similar to preterm children without BPD and term children (52). Nevertheless, in atopic children with very low birth weight, non-specific airway hyperresponsiveness was related to NO levels, derived from central airways (bronchial flux) rather than from peripheral airways (CalvNO). It is likely that the airway hyperresponsiveness is related to the atopic phenotype rather than to the inflammatory process due to prematurity (54). Exhaled breath temperature, another measure of airway inflammation, was significantly lower in BPD survivors than in asthmatic patients (55). Many patients born prematurely, who developed BPD as neonates, are now approaching adulthood. Patients with chronic respiratory symptoms who were born prematurely should undergo comprehensive lung function testing including FeNO measurements. The pathophysiology of BPD is not identical to that of asthma and standard treatment for asthma is therefore ineffective in BPD. FeNO measurements may be useful to differentiate BPD from asthma.

## NO in respiratory infections

The influence of respiratory infections on FeNO has not been studied in detail, although infections of presumed viral origin were early recognized to be associated with an increase in FeNO levels (56, 57). Later studies have indicated that this increase in FeNO is primarily related to rhinovirus infections (58–60), whereas both infections with respiratory syncytial virus (61, 62) and influenza virus (63) seem to be associated with reductions rather than increases in FeNO levels. The reason for increased FeNO levels in rhinovirus infections is poorly studied, but may involve upregulation of interferons causing increased iNOS expression in the bronchial epithelium *via* activation of signal transducer and activator of transcription (STAT)-1 (56). In contrast to rhinovirus infections, bacterial pneumonias are not generally related to increased FeNO levels (64), and neither is the exposure to endotoxin and other bacterial components (65–67). Interestingly, in lung transplant recipients (LTRs), bacterial pneumonias have been shown to be associated with increased FeNO levels, but whether this is a response seen uniquely in these patients is presently unknown.

In summary, FeNO measurements do not seem to be useful in the management of respiratory infections. It is suggested that if a patient with (suspected) asthma reports recent or ongoing symptoms of common cold, the patient should be reexamined with regard to FeNO after a symptom-free interval of at least 3–4 weeks.

## NO in allograft rejections

More than 15 years ago, Silkoff et al. (68) showed increases in FeNO levels within 20 days before or after a suspected acute cellular rejection in LTRs, but not in patients with stable Bronchiolitis Obliterans Syndrome (BOS). In another study, Fisher et al. (69) showed increased FeNO levels in patients with BOS grade 1 (early stage), but not at later stages (BOS grades 2 and 3). Several later studies showed elevated FeNO levels in unstable BOS and in unstable non-BOS patients who later developed BOS (70–72). The increased FeNO levels in these situations were relatively stable and not related to concomitant pulmonary infections which cause only transient (2–3 weeks) increases in FeNO levels in LTRs (73). Neurohr et al. (72) showed a negative predictive value of 97% for FeNO in predicting the development of BOS. Changes in FeNO, more specifically an increase of >10 ppb, had a very good specificity for detecting an acute complication after lung transplantation (74).

The mechanism underlying increased NO formation seen in unstable patients after lung transplantation is not entirely clear. Early on, Gabbay et al. (75) reported that increased iNOS expression in the bronchial epithelium of patients with BOS which positively correlated with FeNO. In contrast to neutrophilic asthma, FeNO has been shown to strongly correlate with neutrophil counts in bronchoalveolar lavage (BAL) fluid of LTRs (72, 75), although the biological link between neutrophilia and increased iNOS expression in bronchial epithelium is presently unknown. Another study showed increased sputum eosinophils and neutrophils in BOS compared to non-BOS patients, which was negatively correlated with lung function parameters (76). A similar relationship to BOS has also been found for both these cell types when measured in BAL fluid (77). These findings may indicate that both eosinophilic and neutrophilic inflammation contribute to the development of BOS. However, a recent study showed that BAL eosinophilia, but not BAL neutrophilia, relates to both mortality and the development of chronic lung allograft dysfunction (BOS and restrictive allograft syndrome) in LTRs (78). Interestingly, one study has suggested that interleukin (IL)-13 is pivotal in the development of BOS (79). Thus, the latter two studies indicate that Th2-driven mechanisms are important for allograft dysfunction in LTRs. These data suggest that FeNO measurement in the management of LTRs warrants further investigation.

## NO in scleroderma and interstitial lung disease

Systemic sclerosis (SSc) is a connective tissue disorder of unknown etiology that is often complicated by pulmonary involvement, with pulmonary arterial hypertension (PAH) and interstitial lung disease (ILD) being the major causes of death. In early studies, it has been presented that FeNO is increased in SSc patients (80), and specifically in association with fibrosing lung disease (81, 82). In contrast, other studies showed lower FeNO levels in SSc patients with ILD compared to patients without ILD (83, 84). Patients with SSc and PAH show relatively low FeNO values suggesting the important role of NO in regulating pulmonary vascular resistance in SSc (82, 85). In some of the early studies reporting increased FeNO levels, higher exhalation flow rates have been used (e.g. 250 mL/s) and, therefore, peripheral airways have probably been sampled to larger extent (82, 83). Later, several studies indeed showed convincing increases in CalvNO levels, whereas no increase in FeNO could be detected (86–89). CalvNO levels are specifically increased in early SSc, suggested to reflect early inflammatory lung involvement in SSc (89). In other studies, increased CalvNO was found to be related to the grade of ILD (87) and alveolitis (90), and has been shown to be a valuable tool for predicting ILD (88) and pulmonary deterioration (91).

So far, application of FeNO measurement in the management of SSc and ILD patients is not supported by data, whereas increased CalvNO concentrations could be used to non-invasively assess the extent of ILD in conditions including SSc.

## NO in pulmonary arterial hypertension

PAH is characterized by an increase in pulmonary arterial pressure and in pulmonary vascular resistance. This is probably because of an imbalance of local vasodilators and vasoconstrictors. For example, elevated levels of endothelin-1, with vasoconstrictor properties, and decreased levels of NO, with vasodilator properties, or NO metabolites have been described (92). Idiopathic PAH (IPAH), that is, PAH without an identifiable cause, is the most commonly studied condition. Decreased FeNO levels have been generally found in patients with PAH and this appears to be especially the case in IPAH (93). The underlying mechanisms might include the lack of substrate, as supplementation with L-arginine increased NO levels (94), and inhibited NOS-related NO production, as higher levels of asymmetric dimethylarginine have been described in IPAH (95).

FeNO levels have been shown to negatively correlate with pulmonary arterial pressure, assessed by echocardiography (96). Recent data in a PAH animal model showed a correlation between treatment-related changes in FeNO and pulmonary arterial pressure (97). Studies in humans are in line with these animal data. An increase in FeNO levels in patients with PAH under treatment was found to be related with the decrease in pulmonary arterial pressure and a prognostic factor for survival (98). Similarly, FeNO levels increased after treatment with endothelin-receptor antagonist (99), prostacyclin (100), or phosphodiesterase 5-inhibition (101). Furthermore, PAH patients who responded to therapy with prostacyclin showed higher baseline FeNO levels (100).

Presently, insufficient evidence exists for recommending routine use of FeNO measurements to predict or follow response to specific PAH-therapy and hence, additional studies need to be conducted.

## NO in hepatopulmonary syndrome

In patients with liver cirrhosis and/or portal hypertension, hepatopulmonary syndrome (HPS) is characterized by an abnormal oxygenation (increased age-corrected alveolar–arterial oxygen difference) due to intrapulmonary vascular dilatations (on contrast-enhanced echocardiography or fractional brain uptake after lung perfusion of technetium-99m macroaggregated albumin lung scanning) (102). The first reports of increased FeNO levels in patients with liver cirrhosis and HPS are those from Cremona et al. (103) and Rolla et al. (104). Both studies showed a significant decrease in FeNO levels and improvement in oxygenation following liver transplantation (103, 105), still the only cure for HPS. It must be stressed that the technique used to collect exhaled air in these studies was markedly different from current recommendations. More recently, by using the technique of multiple flows analysis of NO output, Delclaux et al. (106) demonstrated that the increased FeNO levels previously reported in cirrhosis are of alveolar origin and correlate with the alveolar–arterial oxygen difference. Through additional measurement of NO lung transfer, Degano et al. (107) were able to demonstrate that the increase in CalvNO in cirrhotic patients is due to increased NO output from the alveoli and not due to the decreased lung transfer factor of NO. They showed that in these patients alveolar NO production was associated with hyperdynamic circulatory syndrome, but not with arterial oxygenation impairment. Authors suggest that the lack of correlation between CalvNO and oxygenation impairment observed in their patients may depend on differences in study populations as in previous studies patients had more severe HPS. Alternatively, the relationship between CalvNO and hyperdynamic circulatory syndrome suggests that NO production may be similarly increased in both the pulmonary and the systemic vessels in cirrhotic patients. In conclusion, the above reported observations support the theory that NO plays an important role in the pathophysiology of HPS. However, the enthusiasm for inhibiting NO as therapeutical strategy in HPS has been mitigated by the study of Gomez et al. (108), who reported that inhibition of pulmonary NO-synthase activity (causing acute inhibition of pulmonary NO production) by nebulized NG-nitro-L-arginine methyl ester (L-NAME) had no effect on hypoxemia in patients with HPS.

Whether FeNO or CalvNO measurements can serve to assess the development and the severity of HPS or in the management of this condition awaits further investigation.

## Nasal NO

In parallel to exhaled NO measurements, the value of nasal NO (nNO) samplings in clinical conditions including allergy, primary ciliary dyskinesia (PCD), and CF has been investigated. nNO is produced in the upper airway compartment, especially within the epithelium of the paranasal sinuses, and can be reproducibly sampled (109, 110). Currently, the best-validated sampling method comprises aspiration of nNO at a constant transnasal airflow rate as detailed in the last international recommendations (111). Depending on the sampling flow rates, normal values of nNO are within the range of approximately 100–800 ppb in both children (6–17 years) (112) and adults (113).

In patients with untreated allergic rhinitis (AR), nasal eosinophilia has been shown to be accompanied by increased nNO levels as a result of enhanced iNOS expression within the nasal epithelium (114). Pro-inflammatory stimuli such as intranasal allergen challenge (further) increase nNO in sensitized patients (110), whereas anti-inflammatory therapy reduces nNO (115). However, conflicting data exist on the reproducibility of nNO measurements and its applicability as biomarker of allergic disease. Moody et al. (116) performed weekly nNO measurements in 38 patients with perennial rhinitis during 3 weeks and found weak intrasubject repeatability with no difference in nNO levels between allergic patients and non-allergic individuals. Furthermore, nNO did not reflect disease activity in the allergic patients (116).

In CF, low nNO levels have been reported (117, 118). In some studies, lower nNO levels were associated with increased systemic inflammation, assessed by CRP (119). Furthermore, lower nNO levels were related with colonization with *Staphylococcus aureus* (120). Keen et al. (121) found lower nNO levels in pancreatically insufficient CF patients, with further decreases if their airways were colonized with *Pseudomonas aeruginosa*. In contrast, no relation with *Pseudomonas aeruginosa* was found in another study in CF (118).

In patients with PCD, low nNO levels have been reproducibly measured in several studies (122). Using the handheld device at a nasal flow rate of 5 mL/s, showed a reasonable intrasubject repeatability (coefficient of variation 15%), and an excellent sensitivity (>95%) and specificity (100%) for PCD screening in a validation study (123).

Two recent reports suggest a value for monitoring of nNO in asthma to evaluate asthma control (124, 125). In these studies, nNO levels were negatively related to asthma control both in an adult population enriched with subjects with concomitant rhinosinusitis (124) and in children and young adults with asthma (125).

In summary, although promising in some conditions, especially in PCD and allergy, nNO measurements in routine diagnosis and clinical disease monitoring still awaits validation.

## Future perspectives

For additional future applications of FeNO in other (respiratory) conditions, we need to more precisely define the cut off values and potential confounders. A high or low value in one condition does not necessarily apply in another. Taking smokers with COPD as an example: smoke exposure suppresses FeNO. However, within the group, it is clear that patients responding to ICS have relatively higher FeNO values compared to non-responders (126). Thus a ‘high’ FeNO level in a smoker may be different from a non-smoker, possibly due to down-regulation of both inducible and constitutive NO production. In addition, as some of the potential future applications covered in the present review, such as PCD and CF, prevail in children, it should be highlighted that FeNO cut off values should also be better defined in this population (127).

According to current recommendations, FeNO is measured at a flow rate of 50 mL/s, reflecting NO production from the central airways (1). Measuring NO at flow rates ranging from 10 to 300 mL/s allows the assessing of NO production in the more distal airways (128). Increase in CalvNO indicates uncontrolled Th2-driven inflammation within peripheral airways. Patients already on ICS treatment can thus be switched to ultrafine particles ICS and sometimes the addition of systemic corticosteroids may be effective (129). However, presence of peripheral airways constriction limiting axial diffusion might lead to contradictory results with lower CalvNO levels not excluding distal inflammation (130, 131). Whether CalvNO can be used on an individual basis in the clinical setting needs to be established. Furthermore, the method to be used to estimate CalvNO, as new methods have been proposed (132), as well as the need to assess peripheral obstruction in interpreting CalvNO, highlight a need for consensus on which regression model should be used and how to best estimate NO-contribution from different lung compartments (133). Further evaluation and validation of these parameters is urgently needed to establish the use of relevant inflammatory markers that can be used in clinical practice and as outcome measures in clinical trials.

Partly for economic reasons and limited availability, FeNO is presently measured only within a limited number of clinical settings and only at a few time points. However, as disease activity shows day to day variation, and even variation within the same day, it is desirable to perform more frequent measurements. An increased diurnal variability in FeNO levels was associated with loss of asthma control whereas peak flow variability did not differ between controlled and uncontrolled asthmatics (134). We also need to know whether NO measurements on a regular, preferably daily, basis can help to predict a deteriorating disease beyond asthma. For this purpose, cheaper and easily accessible/applicable home measurement devices are indispensable.

## Conclusions

Still today, the majority of clinical decision making is being based on symptom control and lung function parameters. For the future, we will need reliable and simply measurable biomarkers that can guide our clinical and treatment decisions and help us to monitor treatment effects as well as predicting future deteriorations. Together with blood eosinophils, FeNO is the most commonly used biomarker not only in research but also in clinical practice. In asthma, the clinical importance of FeNO as a marker of Th2-driven inflammation that is likely to respond to ICSs is well-established. However, the role of FeNO in other obstructive airway diseases such as COPD and ACOS remains to be further validated following initial studies showing that increased FeNO levels can predict corticosteroid responsiveness (24, 135). In other respiratory conditions, such as CF, PCD, and PAH, low NO levels (nNO and/or FeNO) may be valuable diagnostic or disease monitoring tools. The feasibility and clinical applicability of partitioning FeNO contributions from peripheral and central airways needs to be further established.
